# An Adaptive Jitter Mechanism for Reactive Route Discovery in Sensor Networks

**DOI:** 10.3390/s140814440

**Published:** 2014-08-08

**Authors:** Juan Antonio Cordero, Jiazi Yi, Thomas Clausen

**Affiliations:** 1 Institute of Information and Communication Technologies, Electronics and Applied Mathematics (ICTEAM), Université catholique de Louvain, Louvain-la-Neuve B-1348, Belgium; E-Mail: j.a.cordero@gmail.com; 2 Laboratoire d'Informatique (LIX), École Polytechnique, Palaiseau 91120, France; E-Mail: thomas@thomasclausen.org

**Keywords:** jitter, reactive routing protocol, route discovery, overhead

## Abstract

This paper analyses the impact of jitter when applied to route discovery in reactive (on-demand) routing protocols. In multi-hop non-synchronized wireless networks, jitter—a small, random variation in the timing of message emission—is commonly employed, as a means to avoid collisions of simultaneous transmissions by adjacent routers over the same channel. In a reactive routing protocol for sensor and ad hoc networks, jitter is recommended during the route discovery process, specifically, during the network-wide flooding of route request messages, in order to avoid collisions. Commonly, a simple uniform jitter is recommended. Alas, this is not without drawbacks: when applying uniform jitter to the route discovery process, an effect called delay inversion is observed. This paper, first, studies and quantifies this delay inversion effect. Second, this paper proposes an adaptive jitter mechanism, designed to alleviate the delay inversion effect and thereby to reduce the route discovery overhead and (ultimately) allow the routing protocol to find more optimal paths, as compared to uniform jitter. This paper presents both analytical and simulation studies, showing that the proposed adaptive jitter can effectively decrease the cost of route discovery and increase the path quality.

## 1. Introduction

Flooding is a key networking operation in wireless multi-hop networks, used for the dissemination of data packets and able to operate in a dynamic network, even in the absence of (or not relying on) accurate information about the network topology. For wireless routing protocols (reactive, proactive or both), flooding is for acquiring or disseminating topological information. In its most basic form, flooding consists of requiring every router in the network to retransmit each received packet exactly once over all of its interfaces. This mechanism leads to the well-known broadcast storm problem [1], a consequence of collisions between concurrent and unnecessary retransmissions of the flooded packet. To counter this problem, more sophisticated mechanisms for efficient flooding have been proposed, implemented and standardized [2–4] to reduce the number of (redundant) retransmissions and, consequently, the amount of packet collisions occurring. Some of these proposals substantially improve the performance of flooding in wireless multi-hop networks.

However, the elimination of redundant retransmissions is not sufficient for completely avoiding packet collisions. Even if no redundant transmissions are present, wireless flooding in self-organized, decentralized networks leads to packet collisions caused by the simultaneous transmission of adjacent routers over the same wireless channel. These collisions constitute an important source of packet losses in wireless flooding.

Different approaches have been explored to address this issue and to minimize its impact in wireless multi-hop networks. Classic MAC (Medium Access Control) collision avoidance mechanisms [5,6] are not suited to current wireless sensor scenarios and are unable to solve all possible cases of collisions (e.g., broadcast or multicast transmissions, collisions between non-neighboring routers). Recent research efforts [7,8] have focused on other alternatives, such as the use of multi-channel assignments in wireless sensor networks. These approaches are able to reduce the problem of collisions in potentially dense networking scenarios, at the cost of adding an additional complexity layer (or relying on previous knowledge of the network topology) and renouncing the semi-broadcast capability of the wireless network.

According to the IETF (Internet Engineering Task Force), the problem of packet collisions in a MANET (Mobile Ad hoc Network) can be further alleviated by introducing jitter (a small, random delay on transmissions) in the network layer. In RFC 5148 [9], the use of jitter is recommended for MANETs and wireless sensor networks as a simple collision avoidance mechanism for routing protocol control traffic, such as periodically scheduled packets or event-triggered packets in the Optimized Link State Routing (OLSR) protocol [10,11] or for MANET-enabled areas in OSPF (Open Shortest Path First) routing [12,13].

### 1.1. Related Work

Subsequent standardization of jitter by the IETF in RFC 5148 [9], its implementation in different routing protocols and research to evaluate and discuss the impact of these techniques in the performance of the protocols making use of them have been undertaken. Friedman *et al.* [14] presented the relationship between the maximal jitter duration and the probability of successful transmission and provided a comparison between different strategies of implementing jitter mechanisms. This paper concluded that implementing jitter at any layer above the IP (Internet Protocol) layer (e.g., at the transport or application layer) brings virtually no benefits. Cordero *et al.* [15] introduced an analytical model for investigating the impact of the standardized jitter mechanism for link-state flooding (which includes jittering and packet piggybacking; see RFC 5148 [9] for details) on network-wide packet dissemination performance. Based on that model, [15] studied and quantified the additional delay incurred, the reduction in the number of transmissions and the effect of jitter in packet size. While this paper focuses on flooding in reactive routing, it borrows model assumptions and terminology from [15] in order to study analytically the impact of jittering. Similarly, [16,17] discuss and present preliminary studies of the issues related to the application of jitter in reactive routing protocols, which are extended and completed in this paper.

This paper studies the use of jitter for improving the performance of reactive (on-demand) routing in wireless multi-hop sensor networks. Reactive routing protocols use flooding to disseminate route request messages in the network until they reach their destination, and the introduction of jitter techniques in route discovery is recommended to minimize systematic collisions. The “delay inversion” effect when using traditional uniform jitter [9] is identified and described, and an adaptive jitter mechanism is then proposed to reduce the route discovery overhead and improve path optimality. Both theoretical and experimental (simulation-based) analyses are performed and discussed; the corresponding results show that the proposed adaptive jittering substantially improves the flooding performance with little cost.

### 1.2. Outline

This paper studies the optimization of jitter mechanisms for route discovery of reactive routing protocols and is organized as follows: Section 2 presents the basics of reactive routing protocols, describes the jitter mechanism for flooding optimization described in RFC 5148 [9] and discusses the main drawbacks of the application of jitter in this form to reactive routing protocols. The paper then introduces and studies the “delay inversion” effect, a specific issue related to the use of standard jittering for route request flooding in reactive protocols. Section 3 describes this effect, proposes an alternative for the jitter distribution (the “adaptive jitter”) and discusses some additional variations of this alternative. Section 4 compares analytically the behavior of the standard (uniform) jitter and the proposed window jitter with respect to the “delay inversion” effect and also examines analytically other side effects of jittering in flooding performance. This is complemented with an experimental performance comparison of the proposed techniques and standard jitter. The characteristics and algorithmic performance of the compared techniques and variants are studied in Section 5 by way of graph simulations. The performance impact of the different jittering techniques, when applied to reactive routing protocols, is studied in Section 6 by way of network simulations. Section 7 discusses the results presented throughout the paper and examines the performance trade-off illustrated in the comparison between the considered jitter techniques and variations. Finally, this paper is concluded in Section 8.

## 2. Reactive Routing Protocols and Application of Jitter

This section introduces the basic operations of reactive routing protocols, as used in some wireless *ad hoc* and sensor networks. Then, the use and impact of jitter on flooding performance are briefly introduced and discussed.

### 2.1. Basic Operations of Reactive Routing Protocols

In a reactive routing protocol, routes are computed on demand, *i.e.*, a router seeks to construct and maintain paths to a destination, only when it has data to deliver to that destination. Consequently, control traffic is generated in response to data traffic only; absent data traffic, no control traffic is generated. Furthermore, as long as data traffic flows along an already established path and no errors are incurred, no control traffic is generated for maintaining the path. Finally, each router is required to maintain the state only for the active paths, on which it is an intermediary, as opposed to, e.g., a complete topology map, as in a classic link state protocol. Unsurprisingly, the main mechanisms in a reactive routing protocol are denoted route discovery and route maintenance.

Reactive routing protocols have been explored in academic literature, as well as have been published as experimental protocols by the IETF: the Ad hoc On-Demand Distance Vector (AODV) protocol, as RFC 3561 [18] in 2003, and the Dynamic Source Routing (DSR) protocol, as RFC 4728 [19], in 2007. Reactive protocols typically find use in wireless *ad hoc* and sensor networks, due to the limited amount of states required in each router. For example, LOADng (Light-weight On-demand Ad hoc Distance-vector routing–Next Generation)—a simplified and extended version of AODV [20]—is the routing protocol component of the G3-PLC (Power Line Communication) ITU-T (International Telecommunication Union Telecommunication Standardization Sector) standard for communication in the “smart grid” [21].

#### 2.1.1. Route Discovery

During route discovery, route request (RREQ) messages are flooded through the network, with each intermediary router forwarding the RREQ also recording the reverse path, *i.e.*, the next hop towards the originator of the RREQ. When the RREQ reaches the sought destination, that destination generates an RREP (route reply), which is unicast along the installed reverse path. RREQ and RREP messages carry a monotonically-increasing sequence number, permitting both duplicate detection and detecting which of the two messages contains the most “fresh” information. The RREQ message carries also the metric semantics of a path, *i.e.*, the “cost” of the path to the originator is recorded and updated by each intermediate router. Two flooding modes are possible: the shortest-delay mode and the shortest-path mode. Depending on the flooding mode, RREQ forwarding and RREP generating rules may be slightly different.

**Shortest delay mode** Routers in the network only forward the first RREQ message received from a given source to a given destination; later arriving RREQs with the same pair (source, sequence_number) will be dropped, even if they advertise better paths than the first RREQ received and forwarded. The requested destination behaves similarly: it generates an RREP only upon the first reception of an RREQ from a given source. Routes discovered in this mode may be suboptimal, but they are acquired with minimal delay.
**Shortest path mode** Routers may forward the first RREQ message received from a given source to a given destination; later arriving RREQs with the same pair (src, sequence_number) will be forwarded only if they advertise better paths than all previously forwarded RREQs with that same (src, sequence_number). The destination behaves similarly, generating an RREP for the first RREQ and then for any subsequent RREQ that advertises a better path than all previously received RREQs with that same (src, sequence_number). Depending on what metric is used for calculating “better”, this can improve the quality of the acquired routes, at the cost of increasing considerably the overhead associated to route discovery processes. This is the most common mode, and used in protocols, like LOADng [20].

#### 2.1.2. Route Maintenance

Route maintenance is performed when an actively used route fails, *i.e.*, when a data packet cannot be delivered to the next hop towards the intended destination. On detecting that a route has failed, a route error (RERR) message is generated. On receiving such an RERR message, the source of the failed data packet can initiate a new route discovery procedure to re-establish connectivity.

### 2.2. Jitter Technique for Route Request (RREQ) Flooding

Simultaneous packet transmissions and, in particular, those performed in reactive protocols during route discovery processes, are likely to cause packet losses in wireless mesh networks, due to collisions between concurrent transmissions of routers having (at least) a common neighbor. In order to prevent or minimize these collisions, RFC 5148 [9] recommends the use of jitter for different cases in which packets may be expected to be sent concurrently. Several well-known reactive protocols (e.g., AODV [18], LOAD [22], LOADng [20]) use or provide support to jitter when flooding RREQ packets over a wireless sensor network.

**Figure 1.**
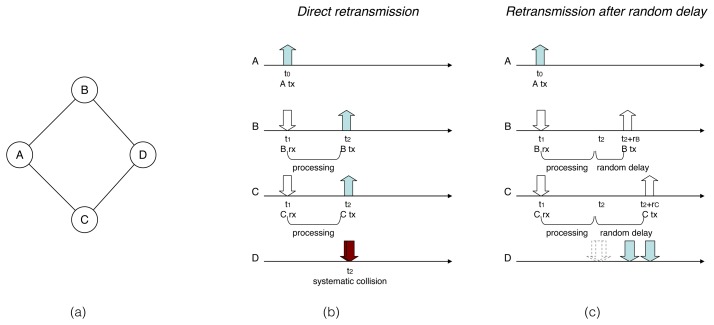
Timeline for packet flooding from *A*: (**a**) Network topology; (**b**)With direct retransmission by *B* and *C*; (c) With retransmission by *B* and *C* after a random delay. The network topology contains four routers {*A*, *B*, *C*, *D*} and four bidirectional links {*AB*, *AC*, *BD*, *CD*}.

Without jitter, a router receiving an RREQ packet to be forwarded retransmits it immediately after processing. As shown by the example in Figure 1, because retransmissions in neighboring routers are triggered by this single event (the reception of the RREQ packet at *B* and *C*), there is a high probability of collision. Instead, when using jitter, every receiving router adds a small, random delay before rebroadcasting the RREQ packet, which dramatically reduces the cases of packet collisions (see Figure 1c).

RFC 5148 [9] recommends that delays are selected following a uniform distribution between zero and a maximum jitter value, *J_m_*. Note that this is the maximum entropy distribution among those assigning continuous jitter values between zero and *J_m_* [23]; the use of this distribution thus maximizes the randomness of the total delay that is incurred by an RREQ packet sent along a certain path.

Other than the prevention of packet collisions from simultaneous transmissions, the use of jitter in flooding has two immediate additional effects:

1. The RREQ flooding, and, therefore, the route discovery, is slowed; and
2. Routers need larger buffers to store packets that have been received, but not yet forwarded.

The trade-off between these drawbacks and the reduction in the probability of collisions, when jitter is uniformly distributed (according to RFC 5148 [9]), can be controlled by way of the length of the jitter interval, *J_m_* [15].

## 3. Delay Inversion and Jitter Optimizations

This section analyses the delay inversion effect, a side-effect of applying uniformly distributed jitter on route request (RREQ) flooding. In order to counter the delay inversion effect, an approach called “window jitter” is introduced, in Section 3.2, applicable for networks using a simple hop count metric. Of course, hop count metrics have their own issues, notably the worst-path-first syndrome, and therefore, Section 3.3 presents a generalization of “window jitter” to non-trivial link metrics.

### 3.1. The Delay Inversion Effect

**Figure 2.**
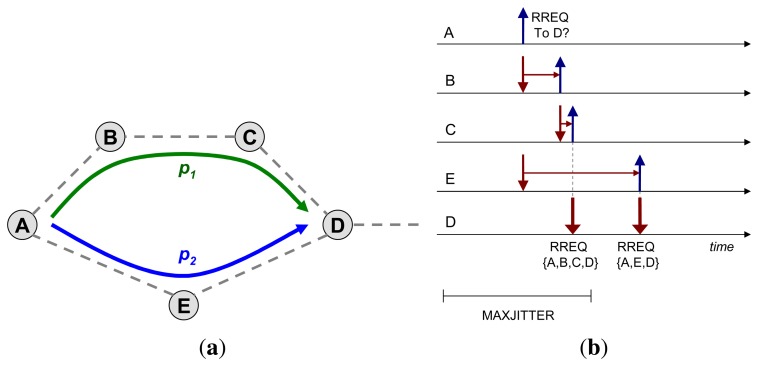
Delay inversion example. Router *A* tries to broadcast an route request (RREQ) message through the network, through paths *p*_1_ and *p*_2_. The RREQ through the longer path {*A*, *B*, *C*, *D*} travels faster than the one through the shorter path {*A*, *E*, *D*}, due to (unfortunate) ittering. (**a**)Topology; (**b**) Example of jitter value assignment for an RREQ from *A* towards *D*.

Consider the topology shown in Figure 2a and assume that router *A* floods an RREQ, in order to discover a route towards *D*. A “trivial” hop count metric is used in this example, *i.e.*, the path with less hops is preferred. Absent jitter, the RREQ would reach *D* through the path *p*_2_ = {*A*, *E*, *D*} faster than it would through the path *p*_1_ = {*A*, *B*, *C*, *D*}; assuming that processing time at each intermediate router, before retransmission, is similar.

Now, if a random delay on retransmission—jitter—is applied at each hop and if that jitter is selected from a uniform random distribution [0, *J_m_*], the message copy sent through the longer path (in terms of the number of hops), *p*_1_, may reach the destination faster than the message copy over *p*_2_; and this, with a non-negligible probability. Figure 2b illustrates this case, and Section 4.1.1 analyzes the probability in further detail.

With reference to Figure 2, consider the transmission of an RREQ packet from *A*, received simultaneously at *B* and *E*. Although the RREQ needs to traverse two hops (*B* and *C*) to reach *D* via *p*_1_ and only one hop (*E*) via *p*_2_, the RREQ sent across *p*_1_ may be received first at D if *j_E_* > *j_B_* + *j_C_*, as shown in Figure 2b.

Router *D* replies to the route request from *A* with an RREP that advertises the (longer-than-shortest) path *p*_1_. When the RREQ, traversing *p*_2_, reaches *D*, *D* replies by generating another RREP that advertises the (shorter) path *p*_2_. This implies that *A* gets, and possibly uses for a certain amount of time, a suboptimal path towards *D* (*p*_1_), and it needs to receive two RREP from *D* in order to learn the optimal path from *A* to *D*. If *D* is not the ultimate destination sought by *A*, then *D* would retransmit two copies of the RREQ—one received via each of the paths *p*_1_ and, then, *p*_2_.

This example illustrates that the use of a uniform random distribution for jitter values when forwarding RREQ packets during route discovery in a reactive routing protocol may lead to cases in which “transmissions over longer paths get first”. This effect is hereafter denominated delay inversion caused by jitter.

Delay inversion is harmful due to at least three undesirable effects:

(i) It increases the (probability of) sub-optimality of reported routes;
(ii) It increases the impact of data traffic forwarded through the network, as a consequence of the use of suboptimal routes; and
(iii) It increases the amount of control traffic: duplicate RREQs forwarded and multiple RREPs generated.

### 3.2. Window Jitter

Window jitter is a small modification of the uniform distribution of jitter, as recommended by RFC 5148 [9]. It introduces a minimum amount of jitter that must be incurred in each hop. Jitter values are, then, instances of a random variable *TJ_W_* ∼ Uniform [*αJ_m_*, *J_m_*], where *α* ∈ (0, 1) and *αJ_m_* is a minimum jitter value. Note that *α* = 0 corresponds to the uniform jitter distribution specified in RFC 5148 [9]; *α* = 1 would imply a deterministic delay (of length *J_m_*). The fact that α ≠ 0 entails that the lower bound for the RREQ delay grows linearly with the length of the traversed path.

Window jitter reduces the randomness and increases the (deterministic) dependency of the total RREQ delay to the length *n* of the traversed path. When assigning jitter values according to the distribution of random variable *J_w_*, the total delay caused by jitter in a path of *n* hops belongs to the interval [*nαJ_m_*, *nJ_m_*] (*α* ≠ 0). The trade-off between randomness and path length deterministic dependence can be controlled by way of parameter *α* ∈ (0, 1): the closer *α* is to one, the more deterministic becomes the total delay of an RREQ packet with respect to the path length.

Under the window jitter distribution, each additional hop in the path traversed by an RREQ packet causes at least an additional delay of *αJ_m_*. Intuitively (see Section 4.1.2 for a more rigorous analysis), this makes it less likely that a larger number of hops is traversed by RREQ packets in a shorter time. The implicit assumption is that “shorter” paths (in the number of hops) are preferable to “longer” paths, considered worse for routing (hop count metric).

### 3.3. Adaptive Jitter for Non-Trivial Metrics

The window jitter principle can be naturally extended to non-trivial link metrics, for instance based on the probability of successful transmission (Expected Transmission Count [24]) or the available bandwidth in the link. This extension of window jitter to link metrics other than hop count is denominated adaptive jitter.

Given a link quality indicator LQ ∈ (0,1) (LQ → 1 for high quality links), jitter values are selected uniformly within the interval [(1 − LQ) *J_m_*, *J_m_*]. This reduces the probability of delay inversion or, equivalently, increases the probability that an RREQ packet is forwarded faster by routers receiving it on better links.

Note that the window jitter distribution presented in Section 3.2 corresponds to the particular case of LQ = 1 − *α* for all available links.

## 4. Analysis

This section examines, analytically, the impact of jitter being applied to RREQ flooding in reactive routing protocols. Two analytic models of jitter distributions are considered: uniform jitter, as specified in RFC 5148 [9], and window jitter as proposed in Section 3.2, for a static *α*. Specifically, Section 4.1 studies the quality of routes discovered throughout the network and the probability that the delay inversion effect occurs when two paths of a different number of hops are available between the requesting source and the requested destination. Following, Section 4.2 proposes simplified models for examining and comparing other side-effects of each uniform and window jitter, in particular, the probability of auto-collisions—two “copies” of the same packet, occurring in the network due to retransmissions and colliding—when flooding an RREQ, and the average number of received-but-not-yet-forwarded RREQs in a router. Appendix A contains the full proofs of the propositions in this section.

### 4.1. Route Sub-Optimality

This section provides a quantitative probabilistic analysis of the delay inversion effect. Let *T_j_* be the random variable for jitter values. The delay caused by jitter in an RREQ message traversing a path of *n* hops, *T*^(^*^n^*^)^, can be then be computed as follows:

(1) $$T^{\left(n\right)}=\sum_{i=1}^{n}T_{j_{i}}$$

Given two paths between a source *X* and a destination *Y*, with lengths *n* and *m*, let *D*^(^*^n^*^,^*^m^*^)^ be the inter-path delay difference, *i.e.*, the difference between jitter delays suffered by an RREQ flooded through two paths between *X* and *Y*, of *n* and *m* hops, respectively. It is a random variable that depends on the random variables for the jitter values in the way shown in Equation (2):

(2) $$\begin{array}{cc} D^{\left(n,m\right)} & =T^{\left(n\right)}-T^{\left(m\right)}=\overset{n}{\underset{i=1}{\text{∑}}}T_{j_{i}}-\overset{m}{\underset{j=1}{\text{∑}}}T_{j_{j}}=\\ & =\overset{n}{\underset{i=1}{\text{∑}}}T_{j_{i}}+\overset{m}{\underset{j=1}{\text{∑}}}\left(-T_{j_{j}}\right) \end{array}$$

The probability that the delay inversion effect occurs in the RREQ flooding corresponds to *Pr*(*D*^(^*^n^*^,^*^m^*^)^ > 0|*_n_*_<_*_m_*), whose expression is detailed in Equation (3):

(3) $$P\left(T^{\left(m\right)}<T^{\left(n\right)}\right)=P\left(D^{\left(n,m\right)}>0\right)=\int_{0}^{\infty }f_{D^{\left(n,m\right)}}(t)dt$$

The probability density function (pdf) of *D*^(^*^n^*^,^*^m^*^)^, *f_D_*^_(_^*_^n^_*^_,_^*_^m^_*^_)_^(*t*), has the following expression:

(4) $$f_{D^{\left(n,m\right)}}(t)=\left(\overset{n+m}{\underset{i=1}{⊗}}f_{T_{j_{i}}}\right)(t+mJ_{m})$$

where ⨂ denotes the convolution.

### 4.1.1. Uniform Jitter

In the case of uniform jitter, *T_j_* ≡ Uniform [0, *J_m_*]. Let *P_U_* and *D_U_* denote the probability of inversion with uniform jitter and the inter-path delay difference with uniform jitter, respectively. Then, the probability of inversion *P_U_* has the expression detailed in Proposition 1.

#### Proposition 1

*The probability of inversion with uniform jitter, *P_U_*, in two paths of n and m, has the following expression:*

(5) $$P_{U}=\frac{1}{(n+m)!}\overset{n+m}{\underset{k=0}{\text{∑}}}\left[{\left(-1\right)}^{k}\left(\begin{array}{c} n+m\\ k \end{array}\right)\times \left({\left(n+2m-k\right)}^{n+m}-{\left(m-k\right)}_{+}^{n+m}\right)\right]$$

**Figure 3.**
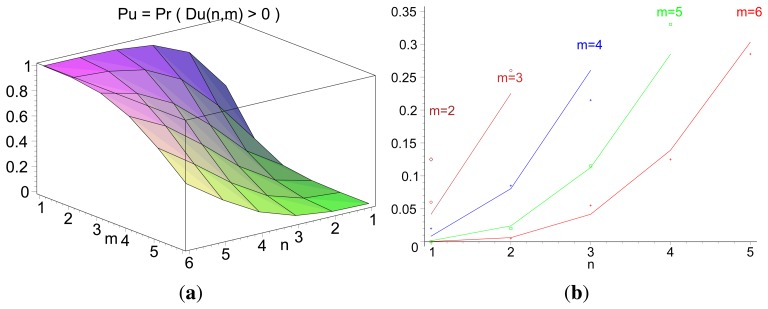
3D and 2D representation of P_U_. (**a**) Theoretical value of [ $P_{U}\equiv Pr(D_{U}^{\left(n,m\right)}>0)$], for 1 ≤ *n* ≤ 6, 1 ≤ *m* ≤ 6; (**b**) Restriction of *p_u_* for *m* = 1, 2, 3, 4, 5, *n* < *m* (theoretical values in lines, simulations in points).

**Figure 4.**
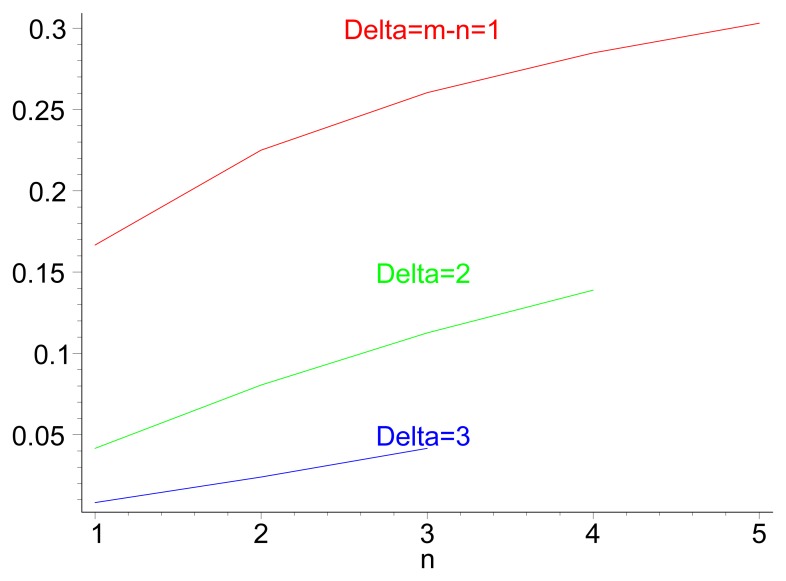
Value of [ $P_{U}\equiv Pr(D_{U}^{\left(n,m\right)}>0)$], depending on Δ = *m* −*n* > 0, for different values of *n*.

Figure 3a illustrates the theoretical values for the probability of inversion for different values *n* and *m*, i.e., the probability that a path of *m* hops performs faster forwarding than a path of length n. Figure 3b displays the same probability for different values of path length *m*, for cases in which *n*< *m*. Both the theoretical values and the results from a discrete-event simulation (each point corresponding to the averaged value over 200 samples) are displayed. Figure 4 illustrates the evolution of the probability of delay inversion between paths with a constant hop difference Δ = *m* − *n* as the absolute length of these paths (in number of hops) increases. Note that both Figures 3b and 4 show bidimensional cuts of the surface presented in Figure 3a; these cuts result from the intersection of this surface with planes *π*_1_ : {*m* = *ct*.} and *π*_2_ : {Δ = *m* − *n*= *ct*.}, respectively.

Expression (5) indicates that the delay inversion occurs, under the conditions specified in RFC 5148 [9], with a significant probability. For the topology presented in Figure 2a, for instance (*n* = 2, *m* = 3), this probability is $Pr(D_{U}^{\left(2,3\right)}>0)=0.225$, implying that the RREQ traversing the longer path will reach the destination first in almost one out of four cases.

Two aspects can be highlighted from this analysis: (i) from Equation (5), the probability of inversion does not depend on the length of the jitter interval, *J_m_*, meaning that it cannot be addressed by modifying the jitter interval length; and (ii) from Figures 3b and 4, the probability of inversion does not only depend on the difference between path lengths, Δ = *m* − *n*, but also on the absolute values of path lengths *n* and *m*: as paths become longer, more random jitter values are assigned to an RREQ message, and it is more likely that delay inversions occur.

The fact that delay inversion is more frequent in long paths (in terms of number of hops) is due to the fact that the range in which total jitter values are possible (adding all per-hop jitter values) has a linearly growing upper bound (*nJ_m_*, where *n* is the path hop length) and a fixed lower bound set to zero. Recall that, while a single uniform jitter value within [0, *J_m_*] has mean $\frac{J_{m}}{2}$ and variance $\frac{J_{m}^{2}}{12}$, the sum of *n* uniform jitter values converges in law (by using the Central Limit Theorem, for *n* → ∞) to a Gaussian distribution with mean $n\frac{J_{m}}{2}$ and variance $n\frac{J_{m}^{2}}{12}$: longer paths (with *n* hops) thus lead to more variant total jitter distributions.

### 4.1.2. Window Jitter

The analysis performed in Section 4.1.1 for *Tj* ≡ Uniform [0, *J_m_*] can be repeated for $T_{j}^{*}\equiv \text{Uniform}[\alpha J_{m},J_{m}]$. The probability density distribution (pdf) of the inter-path delay difference described in Equation (4) thus becomes, for the case of window jitter, $D_{w}^{\left(n,m\right)}$:

(6) $$f_{D_{w}^{\left(n,m\right)}}(t)=\left(\overset{n+m}{\underset{i=1}{⊗}}f_{T_{j_{i}}^{*}}\right)(t+mJ_{m})$$

Without loss of generality, and for the sake of simplicity,it can be assumed in the following that *J_m_* = 1. Then, also using Equation (A1), the pdf detailed in Equation (6) can be expressed in the terms of Proposition 2.

### Proposition 2

*The probability density function of the inter-path delay difference for window jitter*, $f_{D_{W}^{\left(n,m\right)}}(t)$*, has the expression shown in Equation (7):*

(7) $$f_{D_{W}^{\left(n,m\right)}}(t)=\frac{1}{1-\alpha }\frac{1}{(n+m-1)!}\sum_{k=0}^{n+m}{\left(-1\right)}^{k}\left[\left(\begin{array}{c} n+m\\ k \end{array}\right)\times {\left(\frac{t-n\alpha +m}{1-\alpha }-k\right)}_{+}^{n+m-1}\right]$$

*where* (.)*_+_ notation stands for the positive part* (*i.e., z_+_ = z if z ≥ 0, and zero otherwise), for* $\frac{t-\alpha n+m}{1-\alpha }\in [0,n+m]$*, and zero otherwise*.

Therefore, the probability of delay inversion with paths of *n* m, $Pr(D_{W}^{\left(n,m\right)}>0){|}_{n<m}$, can be computed as indicated in Proposition 3.

### Proposition 3

*The expression of* $\text{Pw}\equiv Pr(D_{W}^{\left(n,m\right)}>0)$*corresponds to:*

(8) $$\begin{array}{ll} P_{W}= & \frac{1}{(n+m)!}(\overset{n+m}{\underset{k=0}{\text{∑}}}{\left(-1\right)}^{k}\left(\begin{array}{c} n+m\\ k \end{array}\right){\left(n+m-k\right)}^{n+m}-\\ & -\overset{k*}{\underset{k=0}{\text{∑}}}{\left(-1\right)}^{k}\left(\begin{array}{c} n+m\\ k \end{array}\right){\left(\frac{m-n\alpha }{1-\alpha }-k\right)}^{n+m}) \end{array}$$

*where* $k^{*}={\left\lfloor \frac{m-n\alpha }{1-\alpha }\right\rfloor }_{+}$ for $\alpha \leq \frac{n}{m}$. *P_W_* = 0 *otherwise (i.e., for* $\alpha >\frac{n}{m}$*)*.

**Figure 5.**
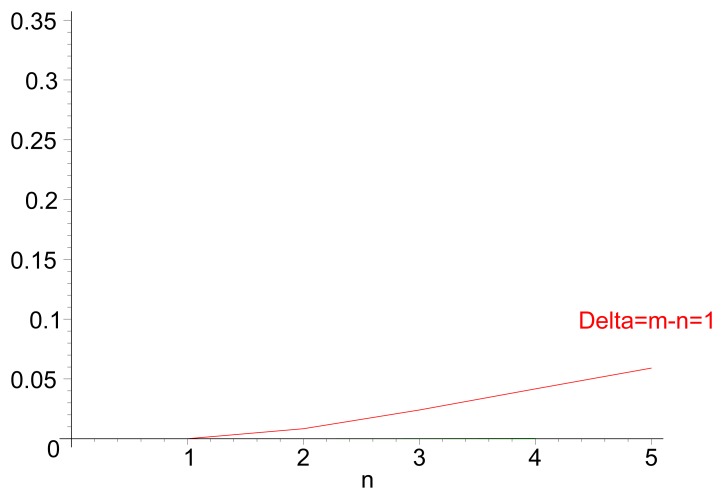
For [ $\alpha =\frac{1}{2}$], 3D and 2D representation of *P_W_*. (**a**) Theoretical value of [ $P_{W}\equiv Pr(D_{W}^{\left(n,m\right)}>0)$], for 1 ≤ *n* ≤ 6, 1 ≤ *m* ≤ 6; (**b**) Restriction of *P_W_* for *m* = 1, 2, 3, 4, 5, *n* < *m* (theoretical values in lines, simulations in points).

Figure 5a,b show the value of $D_{W}^{\left(n,m\right)}$ for different combinations of path lengths *n* and *m*. In the analysis, the hop count metric is considered, *i.e.*, the routes with less hops are more preferred. *α* is set to $\frac{1}{2}$, to have a balance between the randomness of jitter and the “width” of the window to reduce the delay inversion effect.

It can be observed in Figure 5a that the transition from *PW* = 0 to *PW* = 1 (*i.e.*, from situations in which RREQ transmissions over the *n*-path are never faster than those over the *m*-path, to a situation in which they are always faster) is significantly more steep with the modified (generalized) distribution of jitter values than with the distribution of RFC 5148 [9] (see Figure 3a). As the ideal situation would be that $D_{\text{ideal}}^{\left(n,m\right)}=1$ for *n* > *m* and $D_{\text{ideal}}^{\left(n,m\right)}=0$ for *n* < *m*, the use of the modified distribution makes the jitter performance closer to that ideal behavior.

The adaptive jitter for non-trivial metrics is a generalization of window jitter. Note that, under the assumption of the hop count metric (*i.e.*, link quality is constant), in this analysis, window jitter is equivalent to adaptive jitter. Further simulations, presented in Section 5, show experimentally the advantage of adaptive jitter with non-trivial metrics.

**Figure 6.**
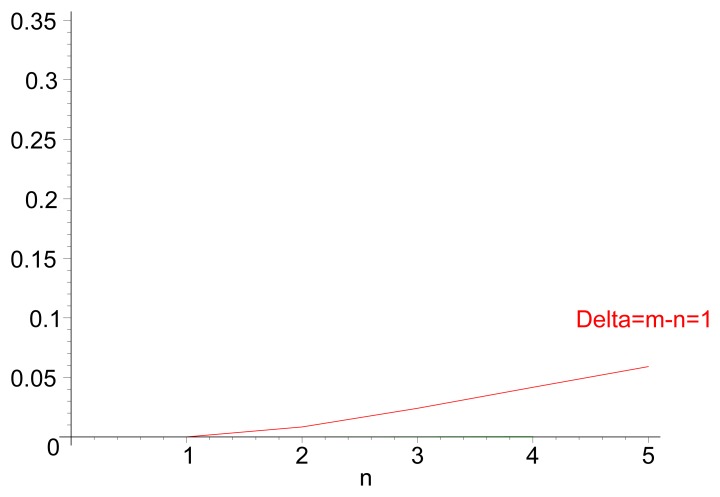
Value of [ $P_{W}\equiv Pr(D_{W}^{\left(n,m\right)}>0)$], depending on Δ = *m* −*n*> 0, for different values of *n*(α = [ $n(\alpha =\frac{1}{2})$]). Traces corresponding to Δ = 2 and Δ = 3 are significantly lower than 0.01 and, thus, are not visible in the picture.

Figure 5b shows the probability of delay inversion for the modified distribution of jitter values, depending on the difference Δ = *m* − *n*, for different values of *n* and *m*. As in Figure 3b, theoretical values (lines) and simulations (points, each of them averaged over 200 samples) are displayed together. It can be observed that the values are substantially lower than those achieved with *T_j_* ∼ Uniform [0, *J_m_*]: for very similar (Δ = *m* − *n* = 1, which is the most frequent case) and long paths (*n* = 5), the probability reduces in a factor of five and stays below 6%; the relative variation becomes still more significant as paths are shorter. The same conclusion can be drawn from the evolution of *P_W_* with respect to Δ = *m* − *n* (Figure 6).

## 4.2. Other Effects

This section examines some additional effects of packet jittering. The impact of standard (uniform) and window jitter in terms of flooding performance and networking requirements is addressed by way of simplified models for flooded packet auto-collisions (Section 4.2.1) and buffering needs in forwarding routers (Section 4.2.2).

### 4.2.1. Auto-Collisions in First Hop

For a packet *p* flooded over a wireless network, auto-collision is the case of two copies of that packet, *p*, colliding; in other words, that *p* “collides with its own alter-ego”.

**Figure 7.**
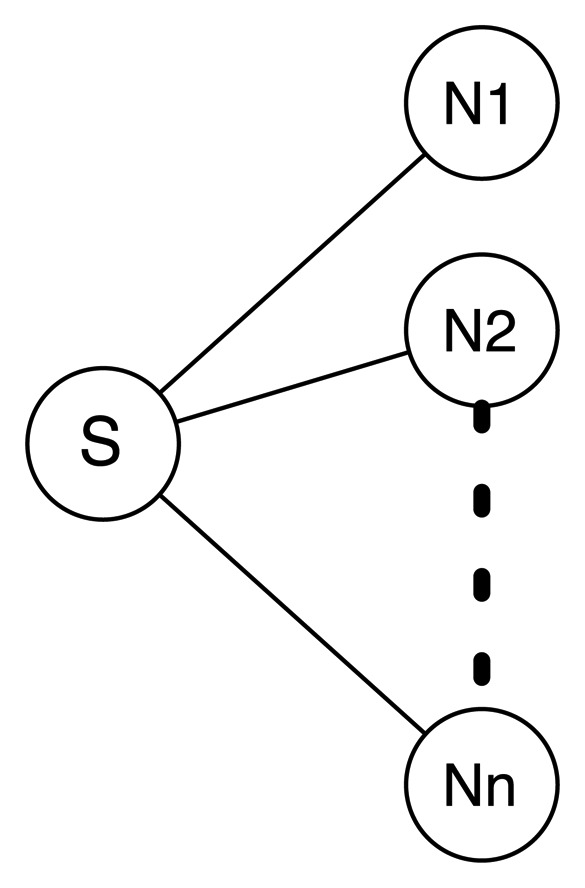
Simple network topology, in which *S* is a neighbor with all of *N*_1_, …, *N_n_* and where all of *N*_1_,…, *N_n_* are neighbors of each other.

Consider the network, depicted in Figure 7, in which a router *S* floods a packet *p* at time *t* = *to* and in which all of *N*_1_, *N*_2_, …, *N_n_* are neighbors. This packet is, then, simultaneously received by the *n* neighbors of *S*, *N*(*S*) = {*N*_1_, *N*_2_,…, *N_n_*} and forwarded (retransmitted) with a random jitter value *T_j_*, distributed according to the different considered mechanisms. For tractability purposes, retransmissions are modeled in this section as a homogeneous Poisson arrival process. In this scenario, there is an auto-collision of *p* in the one-hop neighborhood of *S* when at least two neighbors of *S* retransmit *p* at times *t*_1_ and *t*_2_, such that the inter-arrival time |*t*_1_ − *t*_2_| < *L*, where *L* = *L*(*p*) is the transmission time for packet *p*.

In the case of uniform jitter, retransmission times will be distributed within *t* = *t*_0_ and *t* = *t*_0_ + *J_m_*, where *J_m_* is the upper bound for jitter values; the Poisson rate of retransmissions will be $\lambda_{\text{uniform}}=\frac{n}{J_{m}}$. When using window jitter, retransmissions of a packet *p* received at *t* = *t*_0_ will be confined between *t*_0_ + *αJ_m_* and *t*_0_ + *J_m_* (*α* ∈ (0,1)), that is, no retransmission will occur before *t*_0_ + *αJ_m_*. This implies that the Poisson rate of retransmission will grow to $\lambda_{\text{window}}=\frac{n}{(1-\alpha )J_{m}}$. Lemma 1 describes the probability that the flooding process of packet *p* leads to an auto-collision in the one-hop neighborhood of *S*, with uniform and window jitter.

#### Lemma 1

*If S's neighbors use uniform jitter within* [0, *J_m_*], *the probability of auto-collision in the one-hop neighborhood of S corresponds to Equation (9). If window jitter is used in the same interval, with parameter α, the probability of auto-collision in the one-hop neighborhood of S corresponds to Equation (10):*

(9) $$Pr(\text{auto-collision with uniform jitter})={\left(1-e^{-\frac{nL}{Jm}}\right)}^{n}$$

(10) $$Pr(\text{auto-collision with window jitter})={\left(1-e-\frac{nL}{(1-\alpha )J_{m}}\right)}^{n}$$

**Figure 8.**
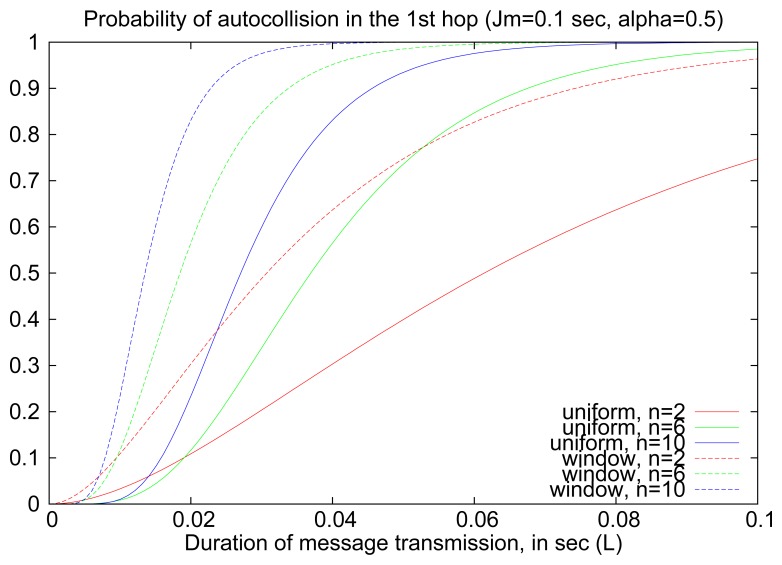
Probability of auto-collision in the first hop.

Figure 8 illustrates the probability of auto-collisions in the first hop of the flooding process (i.e., when an RREQ is retransmitted by routers one hop away from the source) for different sets of parameters (*n*, *J_m_*, *L*). Although the specific values for the probability of auto-collision depend on the chosen values, and, in particular, the relation between *L* and *J_m_*, there are some general trends that can be identified. The use of window jitter leads to more frequent auto-collisions at the first hop, due to the effective reduction of the interval of retransmissions (from [0, *J_m_*] to [*αJ_m_*, *J_m_*]), which implies a more deterministic retransmissions schedule by one-hop neighbors of S. This effect, however, is specific to the first hop and becomes less relevant in multi-hop flooding, as the network has a bigger diameter and routes grow longer, as shown in further experiments (Sections 5 and 6).

### 4.2.2. Buffer Occupancy

When using jitter in a flooding operation, the flooded packets must be stored in buffers in the intermediate routers for the time imposed by the jitter interval. The average occupancy of intermediate routers' buffers is obviously related to the distribution of jitter values.

This section computes the average number of packets to be forwarded by a particular router at time *t* = *t*_0_, for each of the different jittering distributions studied. Assuming a Poisson process with rate λ for packet arrival, the number of packets that arrive (on average) within [*t*_0_ — *J_m_*, *t*_0_) is λ*J_m_*. The probability that one of these is scheduled to be sent later than *t*_0_ is *Pr*(*T̅_t_* + *Tj* > *t*_0_), where *T̅_t_* stands for the random variable of the arrival time (uniformly distributed within [*t*_0_ — *J_m_*, *t*_0_), according to the Poisson model) and *T_j_* is the random variable for jitter value.

Then, the average buffer occupancy *B* has the following expression:

(11) $$B=\lambda J_{m}Pr\left({\bar{T}}_{t}+T_{j}>t_{0}\right)$$

The statistical distribution of *X̅* = *T̅_t_* + *Tj* is determined by the jitter value distribution:

- For the uniform jitter, $Pr(\bar{X}>t_{0})=\frac{1}{2}$.
- For the window jitter (*α* ∈ (0,1)), $Pr(\bar{X}>t_{0})=\frac{1+\alpha }{2}$

Since the use of window jitter entails, for the same value of *J_m_* and the same flooding rate λ, a larger average delay before retransmission than uniform jitter, buffers are expected to store, on average, a *α* additional fraction of packets to be forwarded.

## 5. Graph Simulations and Results

In order to evaluate the performance of the different jitter mechanisms, simulations are performed by way of a Maple-based discrete-event network graph simulator. For simplicity, simulations in this section assume *J_m_* = 1 s (for all types of jitter) and *α* = 0.5 (for window jitter).

### 5.1. Setup

The performance of the three different types of jitter (“standard” jitter, window jitter and adaptive jitter) is evaluated in the shortest-delay mode and shortest-path mode of RREQ flooding (see Section 2.1.1) for different network graph scenarios. The different network scenarios are defined by triplets (*N*, *ρ*, metric), where:

- *N* is the network population (number of routers in the graph);
- *ρ* is the network router density (number of routers per km^2^); and
- “metric” identifies the link metric model: uniform (hop count, in which all available links have cost one) or random (links have a random integer cost from one to 10).

Values for each network profile are averaged over 20 samples, each sample corresponding to a random (static) distribution of routers over the network grid, in which RREQs are sent from a fixed random source to a fixed random destination. Each value related to a distribution corresponds to the average (*µ*) of 10 RREQ flooding procedures simulated between the source and destination; standard deviation (*σ*) intervals around the mean value (*µ* − *σ*, *µ* + *σ*) are also displayed.

The following metrics are used for evaluating the performance of different jitter mechanisms:

- **Number of** (**auto-**)**collisions** Consistently with the definition provided in Section 4.2.1, an auto-collision (denoted as a collision in this section) is counted when a router receives two retransmissions (copies) of the same RREQ, simultaneously.
- **Optimality index** Given a source ***s*** and a destination *d*, the optimality index for a path between ***s*** and *d* is the cost of the shortest path divided by the cost of the discovered path. The path with the index closer to one indicates a better path.
- **Routing overhead** Measured in the number of RREQ or RREP retransmissions.
- **Route discovery delay** In shortest-delay mode, this is the time required in order to discover the first path. In shortest-path mode, it is the time required in order to discover the best path.

### 5.2. Results

This section presents the results of the simulation-based evaluation of “standard” jitter (*i.e.*, uniform jitter according to RFC 5148 [9]), window jitter and adaptive jitter; this for each of the different route discovery modes (shortest-delay and shortest-path), and for different families of network scenarios (fixed grid and constant density) and link metrics (uniform and random link metrics).

#### 5.2.1. Uniform Link Metrics

**Figure 9.**
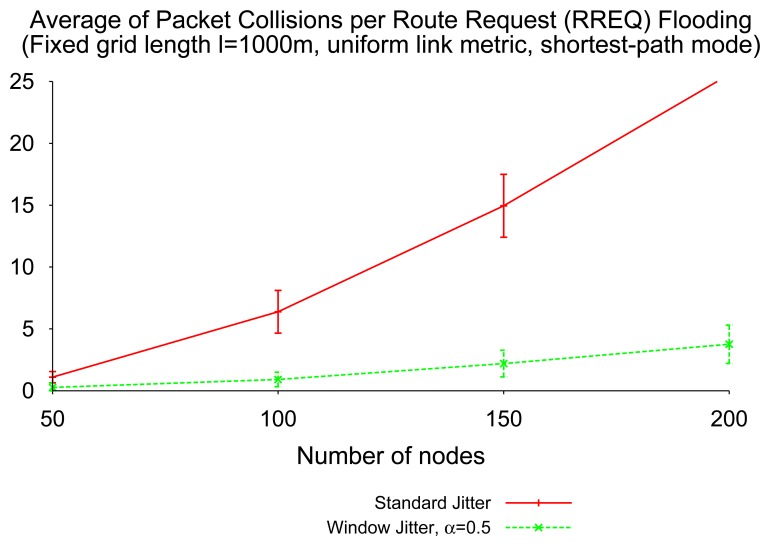
Number of collisions, shortest-path RREQ (Route Request) flooding.

The simulation of the shortest-path mode of route discovery in networks with uniform link cost (hop count) shows that window jitter is able to significantly reduce the number of collisions caused by RREQ flooding, when compared to “standard” jitter. Figure 9 depicts that the collision reduction becomes more relevant as the network density grows.

This reduction is due to the fact that the use of window jitter, when compared to “standard” jitter, increases the probability that the first RREQ received by an intermediate router (or a destination) has traversed the shortest path (according to the metric in use) available, and therefore, no additional RREQ retransmissions need be performed (and no additional route replies need be sent after the first) over a path with better quality than the one previously advertised. The better the quality of the first advertised path, the less RREP control packets involved in a single route discovery process; and the less likely the packet collisions.

**Figure 10.**
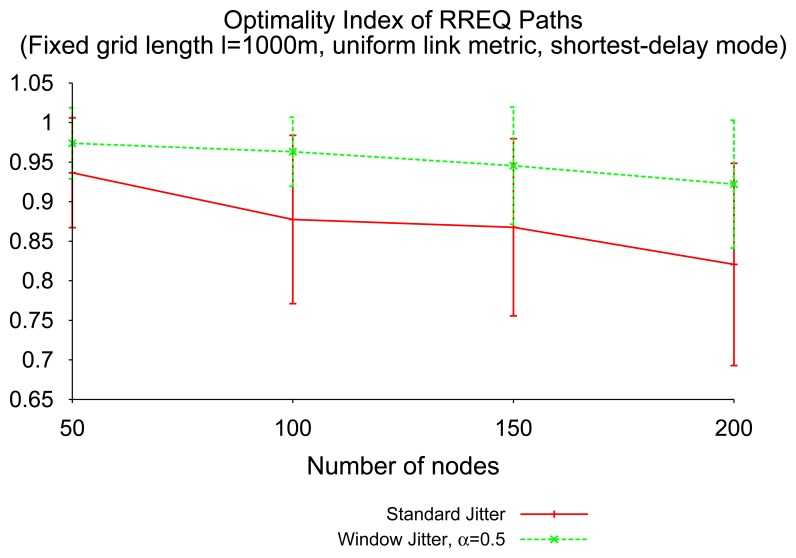
Optimality index, shortest-delay RREQ flooding.

Figure 10 depicts the optimality index of window jitter and “standard” jitter, as a function of network density, when using shortest-delay RREQ flooding. When routers are only allowed to forward the first RREQ received from a given source towards a given destination, the use of window jitter improves significantly the quality of the routes identified through RREQ flooding. This confirms the results from the theoretical analysis of Section 4.1 about the probability of delay inversion in “standard” jitter and window jitter.

As mentioned in Section 3.2, the objective for window jitter is to ensure that the first RREQ that is received, also is the RREQ that has traversed the fewest number of hops and, therefore, represents the better path. Window jitter implicitly assumes a constant link metric, and it is able to provide a significant improvement in the route discovery performance when no more information about link quality is available.

#### 5.2.2. Shortest-Delay Mode over Non-Trivial Link Metrics

**Figure 11.**
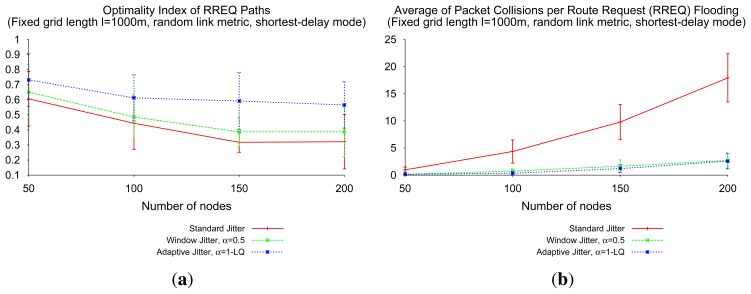
Shortest-delay RREQ flooding. (a) Optimality index; (b) RREP (route reply) packet collisions.

The advantages of window jitter over “standard” jitter is less significant when link metrics are not uniform: the ability to identify better paths by introducing fixed minimum delays (*αJ_m_*) per hop degrades, as depicted in Figure 11. For these non-trivial link metrics, the simulated results show that the use of the adaptive jitter presented in Section 3.3 is more adequate. This is because routers using adaptive jitter can take the actual link metric (e.g., ETX, bandwidth, *etc*.) into consideration, rather than the single presence of these links in the path.

Figure 11a depicts the fact that adaptive jitter clearly outperforms window jitter and “standard” jitter in terms of optimality index. As depicted in Figure 11b for random link quality values, this benefit from the adaptive jitter is compatible with a low level of packet collisions (similar to the level achieved with window jitter and significantly lower than the level achieved with “standard” jitter) in networks with heterogeneous link qualities (*i.e*., non-uniform metrics).

**Figure 12.**
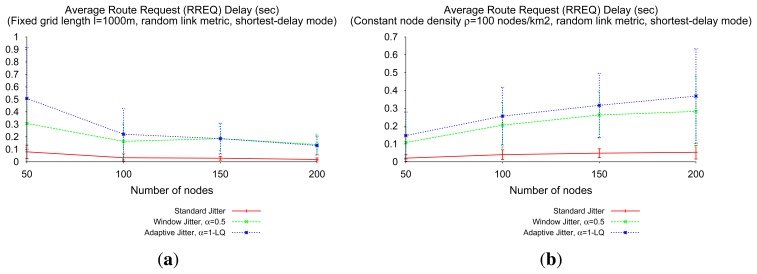
Route discovery delay, shortest-delay RREQ flooding. (**a**) Fixed grid; (**b**) Constant router density.

Discrimination of RREQs based on the quality of traversed links is performed by introducing pre-forwarding delays. This entails a trade-off between RREQ path optimality and RREQ flooding delays, as depicted in Figure 12, for the three types of jitter. In general, the better the path indicated in the first RREQ received by the intended destination, the more delay between the RREQ transmission by the source and its reception in the destination. This can be observed, in particular, for networks of constant router density (Figure 12b). Results from Figure 12a indicate, in addition, that additional delay caused by adaptive jitter with respect to window jitter strongly depends on the network density: as more paths are available to reach the destination (because the network is denser), the heterogeneity of the quality of the involved links in flooding is also higher and the adaptive jitter type allows one to deliver route requests (RREQs) faster, while window jitter cannot reduce the per-hop delay beyond a minimum value *αJ_m_*.

#### 5.2.3. Shortest-Path Mode over Non-Trivial Link Metrics

**Figure 13.**
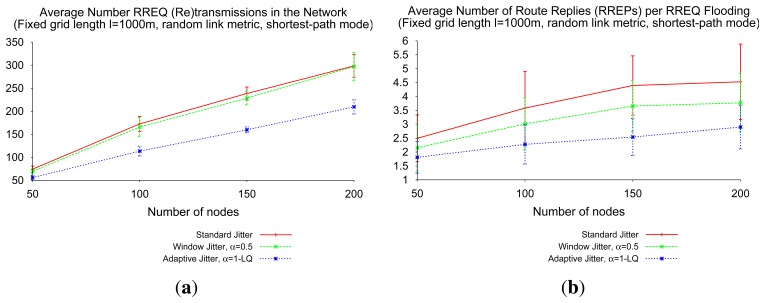
Route overhead per route discovery, shortest-path RREQ flooding. (**a**) The number of RREQ (re)transmissions; (**b**) The number of RREP (re)transmissions.

The use of adaptive jitter in the shortest-path mode of route discovery is also beneficial, although not due to the same reasons (RREQ path quality improvement, mainly) as in the shortest-delay mode. The fact that routers are able to forward RREQs indefinitely, any time that they receive an RREQ with a better route than the last forwarded RREQ, entails that RREQ flooding ideally provides the optimal route between source and destination, if it terminates successfully (without packet losses, collisions or inaccuracies in link quality estimation). However, the shortest-path mode with static jitter (“standard” jitter, window jitter) presents a relevant drawback: as every packet may forward each RREQ several times, and the source may send several RREP to the same destination, the probability of packet collisions and route discovery failure also increases; more significantly for dense networks. Figure 13a,b depicts the evolution of RREQ retransmissions and RREP transmissions per route discovery, when the network density increases. It can be observed that the use of adaptive jitter, by increasing the quality of the firstly-discovered paths, entails a reduction in the number of control packets per route discovery (RREQ retransmissions and route replies) up to 30%, with respect to the static configurations.

**Figure 14.**
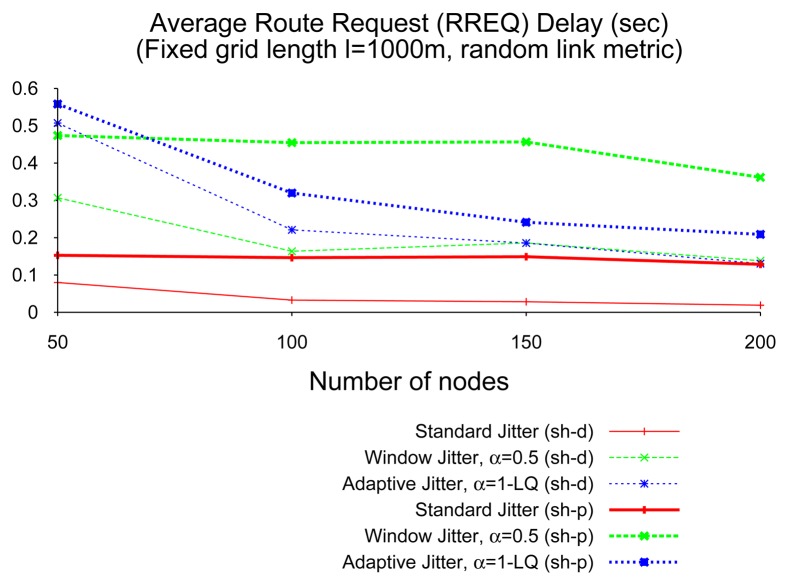
Route discovery delay, shortest-path and shortest-delay RREQ flooding.

Figure 14 depicts average RREQ delays for the different types of jitter when using shortest-path (sh-p) and shortest-delay (sh-d) modes. For any given type of jitter, the delay for the shortest-path mode is always longer or equal to the delay for the shortest-delay mode: in the later, the flooding terminates when the destination receives the first RREQ; in the former, the flooding terminates when the destination receives the RREQ through the best path, which can correspond to the first or to a posterior reception. More interestingly, two observations can be drawn from Figure 14. In the first term, RREQ delay caused by adaptive jitter decreases with the network density (a result consistent with what was depicted in Figure 12), while, in contrast, “standard” jitter and window jitter are present in the shortest-path mode, a roughly constant delay with respect to network density. In the second term, the gap between RREQ delays in shortest-path and shortest-delay modes, i.e., the additional delay caused by reception in the destination of better RREQ packets later than the first, is different for each type of jitter. The adaptive type of jitter has the smallest gap between modes, which is consistent with the previous observation about the quality of first-received RREQs at the destination. The significant difference between modes when using window jitter is another indication, in turn, of the poor performance achieved by this type of jitter in networks with diverse link qualities, as the non-trivial link metrics scenarios considered in this section.

## 6. Network Simulation and Results

Window jitter, proposed in Section 3.2, is implemented, studied and evaluated by way of network simulations of several different network scenarios and parameter configurations. These simulations permit validating the theoretical results obtained in Section 4, as well as the algorithmic evaluation described in Section 5, by considering all network layers, including the transport layer, MAC (Medium Access Control) layer and the physical propagation model.

This section compares the performance of LOADng RREQ flooding when using the original “standard” jitter (*i.e.*, uniform jitter according to RFC 5148 [9]) and the proposed window jitter. Simulations also allow one to identify the networking and jitter elements that have an impact on this performance. Section 6.1 presents the setting of the performed network simulations. Section 6.2 describes the main results obtained in the experiments.

### 6.1. Setting

NS2 (Network Simulator 2) simulation results are presented in Section 6.2. Simulations were made of a field of 1000 × 1000 m, with varying numbers of routers placed randomly, equipped with a 802.11b radio. For the purpose of this study, router mobility was not considered. The metric is based on hop-count.

Each simulation lasts for 100 s. Thirty random routers in the network initiate route discovery to another random destination every two seconds. The number of collisions, average overhead, average route discovery delay and average path length are measured.

Different jitter settings are compared:

- No jitter.
- Standard RFC 5148 jitter, *J_m_* = 100 ms. Jitter is selected within [0,100] ms (mean, 50 ms).
- Standard RFC 5148 jitter, *J_m_* = 200 ms. Jitter is selected within [0, 200] ms (mean, 100 ms).
- Window jitter, $\alpha =\frac{1}{2}$, *J_m_=* 100 ms. Jitter is selected within [50,100] ms (mean, 75 ms).
- Window jitter, $\alpha =\frac{2}{3}$, *J_m_=* 150 ms. Jitter is selected within [100,150] ms (mean, 125 ms).

**Figure 15.**
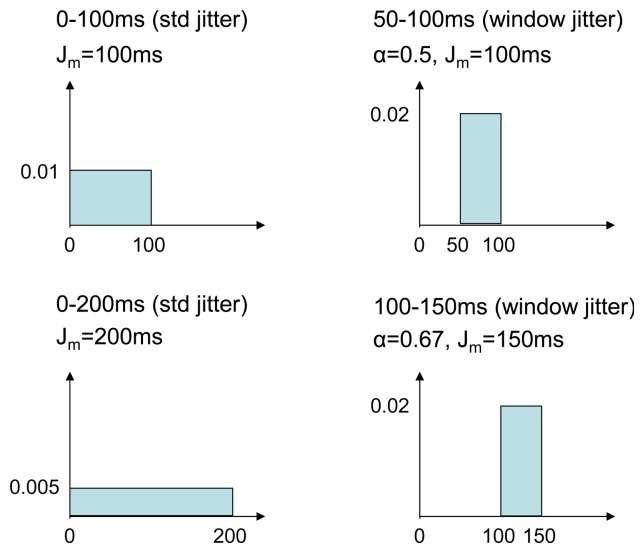
Pdfs for the four examined jitter settings.

Figure 15 shows the probability density function (pdf) for the different jitter settings.

For each of the five settings, the shortest-path and shortest-delay strategies for the RREQ forwarding scheme are evaluated.

### 6.2. Results

This section describes the most relevant results, observed in the performed simulations of various jitter distributions (no jitter, “standard” jitter and window jitter) under shortest-path RREQ forwarding (Section 6.2.1) and shortest-delay RREQ forwarding (Section 6.2.2) configurations.

#### 6.2.1. Shortest-Path RREQ Forwarding

The first observation that can be highlighted from the shortest-path RREQ forwarding results is that the use of “standard” jitter does not have an significant impact on the RREQ flooding performance, when compared with the no-jitter setting; neither in terms of collisions, control overhead or data path length. Differences can be observed, in contrast, between window jitter and “standard” and no jitter.

**Figure 16.**
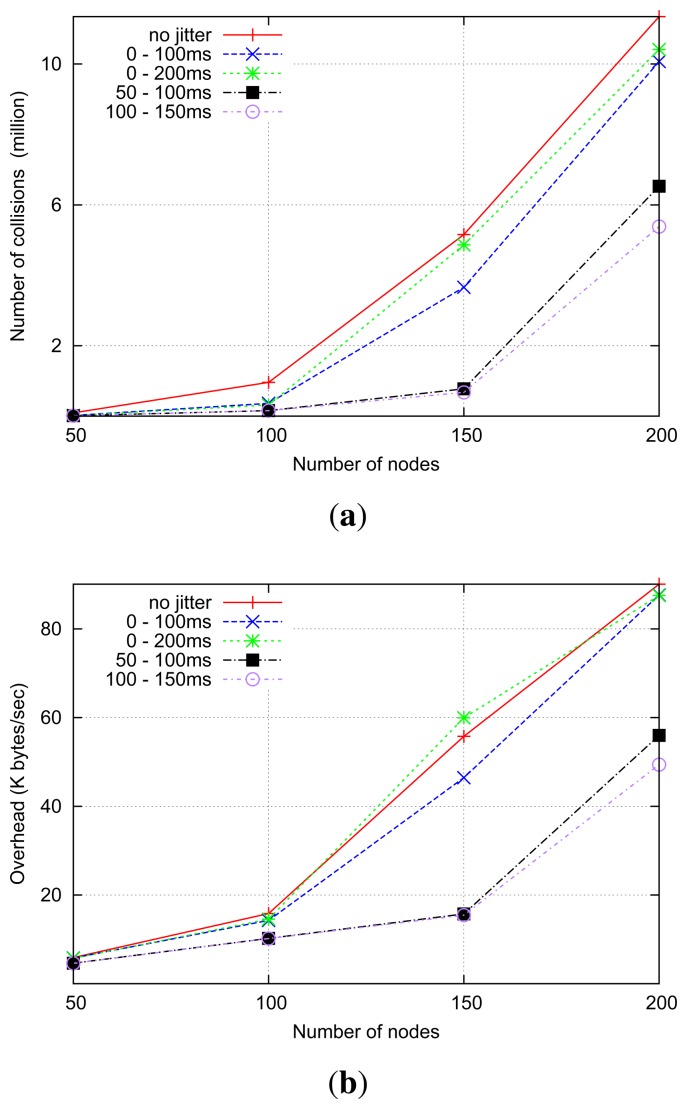
Simulation results of shortest-path RREQ forwarding. (**a**) Number of collisions; (**b**) Average overhead.

Figure 16a shows the number of collisions with a different density of routers. When using no jitter, the highest number of collisions, slightly higher than with “standard” jitter, occurs. This is because adjacent routers are more likely to retransmit received RREQs at the same time. Window jitter (50–100 ms, 100–150 ms) yields significantly fewer collisions, especially in high-density scenarios. This is because the use of the window jitter enables forwarding routers to reduce the number of transmissions (*i.e.*, overhead) by reducing the cases of delay inversion, as shown in Figure 16b.

**Figure 17.**
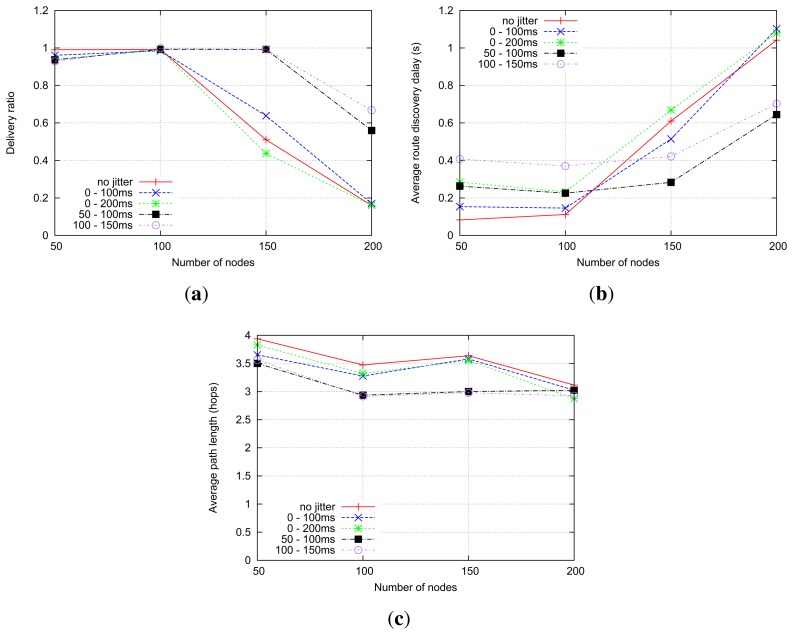
Simulation results of shortest-path RREQ forwarding. (**a**) Data packet delivery ratio; (**b**) The average route discovery delay; (c) Average path length.

Regarding the evolution of route discovery delay (*i.e.*, the time between RREQ transmission and reception of the corresponding RREP) with respect to the network density in the shortest-path RREQ forwarding strategy, depicted in Figure 17b, it is interesting to observe that the impact of the different types of jitter is different for low-density and high-density networks. The average route discovery delay for each type jitter is highly correlated with the mean of the random jitter variable: settings with higher jitter means exhibit higher average delays in the route discovery processes. For denser networks, in contrast, window jitter presents better performance in terms of route delivery delay than “standard” jitter, regardless of the mean value of the jitter random variable. The use of “standard” jitter (or the immediate retransmission of RREQ messages, without jitter) in dense networks leads to an explosion of control traffic when the shortest-path RREQ forwarding strategy is used. This control traffic explosion can be indirectly observed in the evolution of the number of packet collisions in the network, as depicted in Figure 16a, and the data packet delivery ratio, as depicted in Figure 17a. As the control traffic load grows beyond the network capacity, a more significant fraction of transmitted data packets cannot be correctly delivered, even when routes are available, due to the increasing number of packet collisions. By reducing substantially the probability of delay inversion, the use of window jitter distributions improves the quality of the selected routes (see Figure 17c) and allows one to reduce the number of RREPs sent in response to an RREQ. This alleviates the control traffic load of the network and decreases the number of packet collisions, therefore significantly reducing the average delay for route discovery processes.

#### 6.2.2. Shortest-Delay RREQ Forwarding

**Figure 18.**
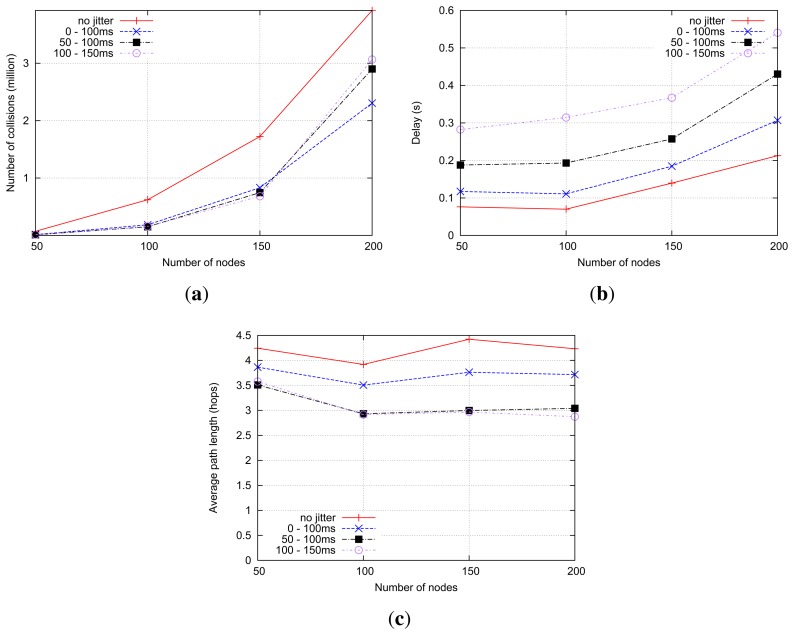
Simulation results of shortest-delay RREQ forwarding. (**a**) The number of collisions; (**b**) The average route discovery delay; (c) Average path length.

As only the first RREQ is forwarded, the network tends to experience the same overhead under the shortest-delay strategy; therefore, the number of collisions is similar regardless of which type of jitter is used, as shown in Figure 18a. The number of collisions in no-jitter settings, in contrast, is obviously higher. In this situation of similar control traffic overhead, those settings using window jitter distributions have an expectedly longer average route discovery delay, as depicted in Figure 18b.

However, because the intermediate routers simply forward the RREQ that arrives first and all of the following RREQs are ignored, the no jitter and “standard” jitter settings got sub-optimal routes. In the meantime, with “window jitter”, a much shorter routes can be explored, as illustrated in Figure 18c. This is more interesting for less time-critical, but power-constrained, networks (such as sensor networks).

## 7. Discussion

Reactive routing protocols rely on flooding, as part of their route discovery process. In wireless networks, flooding incurs the risk of on-the-media collisions and resulting data losses, with the use of jitter on packet (re-)transmissions being the remedy commonly employed for alleviating these losses. The performance of the flooding operation has, as this paper has presented, a direct impact on the quality of the routes discovered. A similar conclusion was found in [25], in which not jitter, but different flooding algorithms were compared for their impact on route quality when used as part of the route discovery process of a reactive routing protocol.

For the purpose of route discovery, the performance of a flooding operation can be evaluated through three main criteria: (1) the resulting quality (path lengths) of discovered routes; (2) the delay incurred when discovering a new route; and (3) the number of collisions caused by route request flooding. Collisions and route quality have a direct impact on the network load: packet collisions may lead to retransmissions, longer paths imply that more transmissions happen than would otherwise be required between a source and a destination. Alas, with respect to jitter, it is not possible to optimize a flooding operation according to all criteria: an infinitely large jitter interval might, for example, eliminate collisions, but would incur unacceptable delays before routes were established. This section summarizes the trade-offs achieved by the different mechanisms proposed and evaluated in this paper.

It is well established that pure flooding (*i.e.*, without jitter) in wireless multi-hop networks entails an unacceptable level of packet collisions. The use of “standard jitter” (*i.e.*, uniform jitter according to RFC 5148 [9] distributed within [0, *J_m_*]) reduces the amount of flooding collisions, at the expense of: (1) slowing down the flooding process; and (2) causing the “delay inversion” effect, as described in Section 3. According to the analysis of Section 4, delay inversion does not depend on the length of jittering time interval (*J_m_*) and becomes more relevant in long paths; that is, in big-diameter networks.

**Figure 19.**
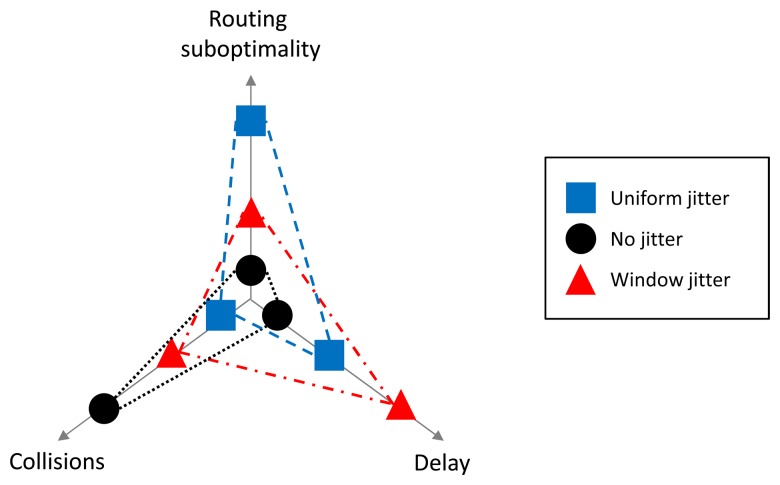
Comparative diagram between no jitter (circle), uniform or standard jitter square) and window jitter (triangle).

Window jitter introduces a minimum non-zero pre-forwarding delay (*αJ_m_*). This dramatically reduces the probability that delay inversion occurs, and thereby an improvement of the quality of discovered routes (in the shortest-delay mode) or a reduction in control overhead and packet collisions (in the shortest-path mode). It also increases the number of collisions in the first hop of flooding of the flooding process (*i.e.*, when an RREQ is retransmitted by routers one hop away from the source), as jitter selection becomes more deterministic. Further evaluation of the performance of RREQ flooding, with “standard” jitter and window jitter, via graph and network simulations (Sections 5 and 6), provide empirical evidence of the route quality improvement with window jitter and shows that the number of collisions along the explored paths is also reduced with this mechanism. Additional collisions in the first hop are thus largely compensated for by the reduction in the number of transmissions farther away; an effect that becomes relevant as the network density increases. Figure 19 provides a qualitative representation of the different trade-offs achieved by the three main variants (no jitter, uniform and window jitter).

Window jitter outperforms standard jitter in hop count networks, but performs poorly when link quality values are heterogeneous. In scenarios in which link quality information is available across the network (for instance, via link quality estimation), adaptive jitter presents clear advantages with respect to the two static configurations (standard and window jitter). In adaptive jitter, pre-forwarding delays depend on the quality of the link through which the packet was received. Not surprisingly, the performance is then better than in static jitter configurations, both in terms of control overhead and route quality. Under the shortest-path mode, route quality is similar for all settings, but adaptive jitter reduces the number of RREQs and RREPs up to 30%. There is still a trade-off between route quality and flooding delay, meaning that adaptive jitter entails additional delays; such a delay becomes negligible as the network density grows.

## 8. Conclusions

Jitter is used for message flooding in wireless multi-hop networks, in order to avoid data losses. It has been shown to be an efficient remedy in proactive routing protocols, such as OLSR [10], and has been recommended for general use by the IETF in RFC 5148 [9].

However, when applied to the route discovery process in reactive routing protocols, using the jitter mechanism prescribed by RFC 5148 [9] has several undesirable side-effects. Observed experimentally, this paper identifies and quantifies analytically the “delay inversion”, which may result in unnecessary control traffic overhead being generated and suboptimal paths to the destination ultimately produced.

Not using jitter is not an option: route discovery relies on the flooding of RREQs, and with intermediate routers immediately retransmitting a received RREQ, collisions will still occur and (RREQ) packets will be lost. Therefore, this paper explores ways to employ jitter on RREQ flooding for reactive routing protocols, while minimizing the impact of delay inversion. In particular, window jitter and adaptive jitter, are compared to “standard” jitter (*i.e.*, jitter according to [9]); and, of course, to naive flooding (*i.e.*, immediate retransmission without jittering). This is not only in terms of incurred delay, but also in terms of avoided collisions, control overhead and quality of discovered routes. Comparisons have been performed by way of both probabilistic analysis and experimental analysis through graph and network (NS2)-based simulations.

The proposed “adaptive jitter” is a simple modification to “standard” jitter, yet despite this simplicity, is able to, very substantially, reduce the probability of experiencing the effect of delay inversion, manifested by sub-optimal routes resulting from the route discovery process. In its most general version, it simply consists of sending flooded messages sooner across better links, which entails a higher probability of the route discovery process resulting in optimal routes. This paper also details the impact of jitter parameters and distribution, on flooding and route discovery performance. Depending on the characteristics of the network, the information available to the routers and the specific route discovery mode, different trade-offs between the main involved parameters (control overhead, route optimality, flooding delay) can be achieved with the proposed jitter distributions.

The results, pretended in this paper, indicate that window jitter (and adaptive jitter, if routers can reliably estimate wireless link quality) is particularly beneficial for reducing overhead in dense networks, in which packet collisions are more frequent and, therefore, delay inversion more likely to happen, due to the the diversity of available paths. These jitter mechanisms thus are interesting for large resource constrained networks, such as in battery-powered mesh and sensor networks, where extraneous transmissions and long paths are particularly harmful to the network lifetime. Future work should see to confirm these results, both through additional analysis, more complex network simulations and real-world experiments. In particular, future work should study the impact of router mobility and wireless channel unreliability on the applicability of window and adaptive jitter.

## Appendix

### A. Proofs

#### Proposition 1

From Equation (2), we have:

$$\begin{array}{cc} D^{\left(n,m\right)} & =T^{\left(n\right)}-T^{\left(m\right)}=\overset{n}{\underset{i=1}{\text{∑}}}T_{j_{i}}-\overset{m}{\underset{j=1}{\text{∑}}}T_{j_{j}}=\\ & =\overset{n}{\underset{i=1}{\text{∑}}}T_{j_{i}}+\overset{m}{\underset{j=1}{\text{∑}}}\left(-T_{j_{j}}\right) \end{array}$$

In the case of $D_{U}^{n,m}$, *T_j_* is a random variable uniformly distributed within [0, *J_m_*].

Therefore, $f_{D_{U}^{n,m}}$ (*t*) can be computed by using the well-known result for the pdf of the sum, *S_n_*, of *n* independent uniform random variables *Uniform* [0, 1] [26]:

(A1) $$f_{s_{n}}(x)=\frac{1}{(n-1)!}\sum_{k=0}^{n}{\left(-1\right)}^{k}\left(\begin{array}{c} n\\ k \end{array}\right){\left(x-k\right)}_{+}^{n-1}$$

Note that f_Sn_(*x*) = 0 for *x* > *n* and *x* < 0. Notation *z*_+_ denotes the positive part of *z*, *i.e.*, *z*_+_ = *z* if *z* > 0, *z*_+_ = 0 otherwise. Then, the probability of delay inversion between two paths with lengths *n* and *m* (*n* < *m*), becomes $Pr(D_{U}^{\left(n,m\right)}>0){|}_{n<m}$. The expression of $P_{U}\equiv Pr(D_{U}^{\left(n,m\right)}>0)$ is computed in Equation (A2):

(A2) $$P_{U}=\begin{array}{l} \frac{1}{(n+m)!}\overset{n+m}{\underset{k=0}{\text{∑}}}[{\left(-1\right)}^{k}\left(\begin{array}{c} n+m\\ k \end{array}\right)\times \\ \times \left({\left(n+2m-k\right)}^{n+m}-{\left(m-k\right)}_{+}^{n+m}\right)] \end{array}$$

#### Proposition 2

(A3) $$f_{D}(t)=\left(\overset{n}{\underset{i=1}{⊗}}f_{T_{j_{i}}^{*}}(t)\right)⊗\left(\overset{m}{\underset{i=1}{⊗}}f_{\left(-T_{j_{i}}^{*}\right)}(t)\right)$$

(A4) $$\{\begin{array}{l} f_{T_{j_{i}}^{*}}(t)=\frac{1}{1-\alpha }p\left(\frac{t-\alpha }{1-\alpha }\right)\\ f_{\left(-T_{j_{i}}^{*}\right)}(t)=\frac{1}{1-\alpha }p\left(\frac{-t-\alpha }{1-\alpha }\right)=\frac{1}{1-\alpha }p\left(\frac{t+1}{1-\alpha }\right) \end{array}$$

where:

p(t)=Heaviside(t)−Heaviside(t−1)

Then, Equation (A3) becomes, by using Equation (A4):

(A5) $${\left(1-\alpha \right)}^{n+m-1}\times \frac{1}{{\left(1-\alpha \right)}^{n+m}}\left(\overset{n+m}{\underset{i=1}{⊗}}p\right)\left(\frac{t-n\alpha +m}{1-\alpha }\right)$$

Note that $\left({⊗}_{i=1}^{n+m}p\right)$

(*x*) is the pdf of the sum of *n* + *m* uniform independent random variables within [0,1]. Therefore, by applying Equation (A1), Equation (A5) becomes:

(A6) $$\frac{{\left(1-\alpha \right)}^{-1}}{(n+m-1)!}\sum_{k=0}^{n+m}{\left(-1\right)}^{k}\left(\begin{array}{c} n+m\\ k \end{array}\right){\left(\frac{t-n\alpha +m}{1-\alpha }-k\right)}_{+}^{n+m-1}$$

#### Proposition 3

From the definition of probability, $Pr(D_{W}>0)=\int_{0}^{\infty }f_{D_{W}}(x)dx$, which is:

(A7) $$\begin{array}{cc} P_{W} & =\int_{0}^{\infty }\frac{dt\sum_{k=0}^{n+m}{\left(-1\right)}^{k}\left(\begin{array}{c} n+m\\ k \end{array}\right){\left(\frac{t-n\alpha +m}{1-\alpha }-k\right)}_{+}^{n+m-1}}{(1-\alpha )(n+m-1)!}=\\ & =\frac{\sum_{k=0}^{n+m}{\left(-1\right)}^{k}\left(\begin{array}{c} n+m\\ k \end{array}\right)\int_{0}^{\infty }dt{\left(\frac{t-n\alpha +m}{1-\alpha }-k\right)}_{+}^{n+m-1}}{\left(1-\alpha \right)\left(n+m-1\right)!} \end{array}$$

The integrand of Equation (A7) is zero when $\frac{t-n\alpha +m}{1-\alpha }>n+m\Leftrightarrow t>n-m\alpha$, and *n* − *mα* > 0 (see Proposition); therefore, Equation (A7) can be rewritten as follows:

(A8) $$P_{w}=\frac{\sum_{k=0}^{n+m}{\left(-1\right)}^{k}\left(\begin{array}{c} n+m\\ k \end{array}\right)\int_{0}^{M}dt{\left(\frac{t-n\alpha +m}{1-\alpha }-k\right)}_{+}^{n+m-1}}{(1-\alpha )(n+m-1)!}$$

where *M* ≡ max {*0*, *n* − *mα*}. Consider the integral $I_{0}\equiv \int_{0}^{M}dt{\left(\frac{t-n\alpha +m}{1-\alpha }-k\right)}_{+}^{n+m-1}$. The integrand of *I*_0_ is non-zero when:

(A9) $$\frac{t-n\alpha +m}{1-\alpha }-k>0\Rightarrow t>k(1-\alpha )+n\alpha -m\equiv t_{0}$$

and zero otherwise. Therefore, integration limits of *I*_0_ can be modified as follows:

(A10) $$I_{0}=\int_{\max\{0,t_{0}\}}^{\max\{0,n-m\alpha \}}dt{\left(\frac{t-n\alpha +m}{1-\alpha }-k\right)}^{n+m-1}$$

*t*_0_ depends on *k*. It is worth observing that:

- *t*_0_ <*n*− mα, ⇔*k* : 0 < *k* < *n* + *m*. And *n* −*mα* > 0 if $\alpha >\frac{n}{m}$.
- *t*_0_ > 0 ⇔ *k*(1 − *α*) + *nα* −*m* > 0, for *k*_min_ = 0 and *k*_max_ *= n* + *m*. This implies that $\alpha >\frac{n}{m}$ and $\alpha >\frac{m}{n}$.
- For $\alpha >\frac{n}{m}$, $\alpha >\frac{m}{n}$, there is *a* $k^{*}={\left\lfloor \frac{m-n\alpha }{1-\alpha }\right\rfloor }_{+}\geq 0$ such that *t*_0_ > 0, ∀*k* > *k**.

Which implies that *I*_0_ can be expressed as:

(A11) $$I_{0}=\int_{\max\{0,t_{0}\}}^{\max\{0,n-m\alpha \}}dt{\left(\frac{t-n\alpha +m}{1-\alpha }-k\right)}^{n+m-1}$$

Then, Equation (A8) can be computed as follows:

(A12) $$P_{w}=\{\begin{array}{ll} 0 & ,\alpha >\frac{n}{m}\\ \frac{1}{(n+m)!}\sum_{k=0}^{n+m}\left[{\left(-1\right)}^{k}\left(\begin{array}{c} n+m\\ k \end{array}\right)\times \times \left({\left(n+m-k\right)}^{n+m}-{\left(n+\frac{m}{1-\alpha }\right)}^{n+m}\right)\right] & ,\frac{m}{n}<\alpha \leq \frac{n}{m}\\ \frac{1}{(n+m)!}\left(\sum_{k=0}^{n+m}{\left(-1\right)}^{k}\left(\begin{array}{c} n+m\\ k \end{array}\right){\left(n+m-k\right)}^{n+m}-\sum_{k=0}^{k^{*}}{\left(-1\right)}^{k}\left(\begin{array}{c} n+m\\ k \end{array}\right){\left(\frac{m-n\alpha }{1-\alpha }-k\right)}^{n+m}\right) & ,\alpha \leq \frac{n}{m},\frac{m}{n} \end{array}$$

##### Lemma 1

In the described scenario (see Figure 7) with *n* neighbors (*n* > 1), there is an auto-collision if the minimum inter-arrival time is smaller than the transmission time for a packet, *L*(*p*). Since inter-arrival times *δ_i_* = |*Tj_i_* − *T_ji+_*_1_| are independent, according to Poisson's hypothesis, we have:

$$\begin{array}{ll} Pr\left(\text{auto}-\text{collision}\right) & =P(\underset{0<i\leq n}{\min}\{|T_{j_{i}}-T_{j_{i+1}}|\}<L)=\\ & =\prod_{0<i\leq n}Pr(\{|T_{j_{i}}-T_{j_{i+1}}|\}<L)=\\ & =Pr{\left(\{|T_{j_{i}}-T_{j_{i+1}}|\}<L\right)}^{n} \end{array}$$

The distribution of *δ_i_* is exponential with rate λ; therefore:

(A13) $$\begin{array}{ll} Pr\left(\text{auto}-\text{collision}\right) & =Pr{\left(\{|T_{j_{i}}-T_{j_{i+1}}|\}<L\right)}^{\text{n}}=\\ & ={\left(\int_{0}^{L}\lambda e^{-\lambda x}dx\right)}^{n}=\\ & ={\left(e^{-\lambda L}-1\right)}^{n} \end{array}$$

Expression (A13) becomes, for uniform jitter $\left(\lambda_{\text{uniform}}=\frac{n}{J_{m}}\right)$:

$$Pr{\left(\text{auto}-\text{collision}\right)}_{\text{uniform}}={\left(e^{-\frac{n}{J_{m}}L}-1\right)}^{n}$$

Additionally, for window jitter, with parameter *α* $\left(\lambda_{\text{window}}=\frac{n}{(1-\alpha )J_{m}}\right)$:

$$Pr{\left(\text{auto}-\text{collision}\right)}_{\text{uniform}}={\left(e^{-\frac{n}{(1-\alpha )J_{m}}L}-1\right)}^{n}$$
